# Emerging of a new CD3^+^CD31^H^CD184^+^ tang cell phenothype in Sjögren’s syndrome induced by microencapsulated human umbilical cord matrix-derived multipotent stromal cells

**DOI:** 10.3389/fimmu.2023.1095768

**Published:** 2023-03-14

**Authors:** Pia Montanucci, Onelia Bistoni, Matteo Antonucci, Teresa Pescara, Alessia Greco, Giuseppe Basta, Elena Bartoloni, Roberto Gerli, Riccardo Calafiore

**Affiliations:** ^1^ Laboratory for Endocrine Cell Transplants and Biohybrid Organs, Department of Medicine and Surgery, University of Perugia, Perugia, Piazzale Gambuli, Italy; ^2^ Division of Rheumatology, Perugia Hospital, Perugia, Piazzale Giorgio, Italy; ^3^ Rheumatology Unit, Department of Medicine and Surgery, University of Perugia, Perugia, Piazzale Giorgio, Italy

**Keywords:** Sjogren’ s syndrome, human multipotent stromal cells, angiogenetic T cells, B regulatory cells (B10), CD31, immunoprotection

## Abstract

**Background:**

Sjögren’s syndrome (SS) is an autoimmune disease hallmarked by infiltration and destruction of exocrine glands. Currently, there is no therapy that warrants full recovery of the affected tissues. Umbilical cord-derived multipotent stromal cells, microincapsulated in an endotoxin-free alginate gel (CpS-hUCMS), were shown to modulate the inflammatory activity of PBMCs in SS patients *in vitro*, through release of soluble factors (TGFβ1, IDO1, IL6, PGE2, VEGF). These observations led us to set up the present study, aimed at defining the *in vitro* effects of CpS-hUCMS on pro- and anti-inflammatory lymphocyte subsets involved in the pathogenesis of SS.

**Methods and results:**

Peripheral blood mononuclear cells (PBMCs) upon collection from SS patients and matched healthy donors, were placed in co-culture with CpS-hUCMS for five days. Cellular proliferation and T- (Tang, Treg) and B- (Breg, CD19^+^) lymphocyte subsets were studied by flow cytometry, while Multiplex, Real-Time PCR, and Western Blotting techniques were employed for the analysis of transcriptome and secretome. IFNγ pre-treated hUCMS were assessed with a viability assay and Western Blotting analysis before co-culture. After five days co-culture, CpS-hUCMS induced multiple effects on PBMCs, with special regard to decrease of lymphocyte proliferation, increase of regulatory B cells and induction of an angiogenic T cell population with high expression of the surface marker CD31, that had never been described before in the literature.

**Conclusion:**

We preliminarily showed that CpS-hUCMS can influence multiple pro- and anti-inflammatory pathways that are deranged in SS. In particular, Breg raised and a new Tang phenothype CD3^+^CD31^H^CD184^+^ emerged. These results may considerably expand our knowledge on multipotent stromal cell properties and may open new therapeutic avenues for the management of this disease, by designing *ad hoc* clinical studies.

## 1 Introduction

Sjögren syndrome (SS) is a systemic autoimmune disorder consisting of chronic inflammation of exocrine glands which leads to impairment of their secretory function and tissue damage. The disease course is rather mild in many patients, although a subgroup of subjects may develop severe extra-glandular effects. Quite often, currently available management options in these cases are ineffective (1–3). Therefore, identification of further pathogenic mechanisms that could be instrumental to therapeutic alternatives would be more than desirable.

Mesenchymal stem cells (MSC) exhibit natural propensity to exert anti-inflammatory and immunomodulatory effects. In particular, post-partum Wharton’s jelly-derived human adult mesenchymal stem cells (hUCMS (4), one of the “youngest” available mesenchymal stem cells, have been deemed to positively condition the immune system by both, reducing pathogenic T-cell subsets and potentiating their regulatory counterparts. In fact, *in vitro* overnight pre-treatment of hUCMS with the pro-inflammatory cytokine interferon-gamma (IFN-γ), induced the expression of molecules involved in the tolerogenic process, at the fetal-maternal interface, like indoleamine 2,3-dioxygenase 1 (IDO1), involved in the tryptophan catabolism, and led to an increase three classes of HLA: HLA-E, HLA-F and HLA-G. In particular, HLA-G5 is non-classical histocompatibility antigen complex, which was reported to contribute to hUCMS tolerogenic properties (5, 6). These immune-active features favored application of MSC-based cell therapy to an array of autoimmune diseases including Systemic Lupus Erythematosus (SLE) (7), type 1 diabetes mellitus (T1D) and Sjögren Syndrome (SS), with the purpose of restoring a state of acquired immune tolerance (8–10).

To fully exploit hUCMS properties, we have enveloped them in our highly performing, and biocompatible alginate (AG)-based microcapsules (11) that physically separate the cells from the host’s immune system. AG derivatives still represent the most successful polymeric material associated with high biocompatibility and favorable porosity/permeability properties for microencapsulation of live cells. Meticulous purification technologies of the raw AG product (Process for the ultrapurification of alginates, Patent no. WO 2009093184 A139), originally extracted from brown seaweeds, have enabled fulfillment of regulatory criteria for human application.

We had preliminarily showed that IFN-γ-licensed microencapsuled hUCMS might positively impact on the immune system by expanding Tregs, *in vitro*, derived from patients with SS and T1D (9, 10). Additionally, we proved that microencapsulated hUCMS transplanted in NOD mice with recent-onset autoimmune diabetes restored normoglycemia (12). This outcome was ascribed to hUCMS-related both, immunomodulatory effects on regulatory T-cell subsets (Tregs), and paracrine action on islet cells.

The purpose of the present work was to further investigate the effects of *in vitro* co-culture of SS patient-derived PBMCs with microencapsulated hUCMS, on different T lymphocyte subsets (having Treg been investigated previously) (9) and also on B-cell subsets with special regard to regulatory Breg.

## 2 Materials and methods

### 2.1 Demographics and PBMC donor patient inclusion criteria

A cohort of patients aged 18 years or older with a diagnosis of SS according to ACR/EULAR 2016 criteria (13) were consecutively enrolled. As expected, all patients were females due to the high prevalence of the disease SS in female sex. A cohort of 6 age- and sex-matched healthy volunteers with a negative history for autoimmune/allergic diseases were included as normal controls. Exclusion criteria were: positive history for autoimmune/allergic diseases different from SS, ongoing immunosuppressive and glucocorticoid therapy, ongoing infectious disorders of any etiology, or positive history for infectious illness within 30 days prior to enrollment. Patients and healthy controls were consecutively enrolled and the study design did not require randomization or blinding. The study was approved by the local ethical committee (CEAS) and a written informed consent was obtained from all the selected subjects in accordance with the Declaration of Helsinki.

### 2.2 PBMC isolation, co-culture and phenotypic analysis

30mL of heparinized peripheral blood was collected from each subject, the latter of which was used for CBC analysis. PBMCs were isolated by standard gradient separation on Lymphoprep™. For cell proliferation studies, the cells were labeled by CFSE Cell Division Tracker Kit (BioLegend) following the manufacturer’s instructions. PBMCs were co-cultured for 5 days with CPS-hUCMS after priming o/n the preparations with IFN-γ (2400U/10^6^ cells). Unstimulated, and anti-human CD3 soluble (clone HIT3α, BioLegend) 1µg/mL-triggered PBMCs served for negative and positive controls, respectively. PBMCs : CPS-hUCMS ratio was 50:1. (6x10^3^ hUCMS:3x10^5^ PBMCs) in 300µL of complete CMRL medium. Cytofluorimetric assessment was performed using fluorochrome-conjugated monoclonal antibodies: CD3-PeCy7, CD3-FITC, CD3-eFluor450, CD4-PE, CD4-PeCy5.5, CD8-PeCy7, CD8-BV605, CD19-APC, CD24-PE, CD25-FITC, CD28-FITC, CD31-PE, CD38-PeCy7, CD45RA-BV510, CD184-APC, FOXP3-PE, IL10-AF488, IL17A-AF647. Antibodies were provided by Thermo Fisher Scientific and BD Pharmingen. As for IL17A, the cells were pre-treated with protein transport inhibitor cocktail (500x) (Thermo Fisher Scientific) following vendor’s recommendations. Before intracellular staining, the cells were fixed with 0.5% formaldehyde and permeabilized with 0.1% saponin. All cytofluorimetric assays were performed on the FACSCalibur™ Flow Cytometer with CellQuestPro™ (BD Bioscience) software and analyzed by FlowJo (RRID : SCR_008520) software (Tree Star Inc.).

### 2.3 hUCMS procurement, isolation and culture

hUCMS isolation procedure from post-partum umbilical cords, obtained by caesarean section or natural childbirth, followed our established method (4). At the end of the isolation/purification process, the cells were seeded, at a concentration of 6000 to 8000/cm^2^ per culture flask and incubated at 37°C in humidified 95% air. Cell expansion throughout 80% confluence was achieved by treatment with 0.05% trypsin/EDTA (Gibco, Invitrogen, Milan, Italy) for 3 minutes at 37°C.

### 2.4 AG procurement and purification for microencapsulation

Powdered alginate (AG) was purchased from Monsanto-Kelco (San Diego, CA) featuring the following properties: molecular weight=120-190 kDa; main AG polymeric patterns: mannuronic acid, M fraction (FM) 61%); and guluronic acid G fraction (FG) 39%. AG ultra-purification was conducted in-house under good laboratory practice (GLP), according to methods developed in our laboratory ((Process for the ultra-purification of alginates, Patent no. WO 2009093184 A139); endotoxin levels were <0.5 EU/mL, protein content <0.45%, while viscosity was 100-300 cps.

### 2.5 Microencapsulation of hUCMS

Briefly, hUCMS were thoroughly mixed with a 1.8% AG solution, until a homogenous suspension was obtained. The cell/AG ratio was 3x10^6^ hUCMS/1.2 ml AG. The suspension was then mechanically extruded through a microdroplet generator (11), and the AG/cell microdroplets were collected on a 1.2% CaCl_2_ bath. Upon coating with 0.05% poly-L-ornithine chloride (Sigma-Aldrich), the microbeads were partially de-gelled by 55mM sodium citrate for 10 minutes at r.t. The obtained microcapsules were finally coated with 0.1% AG, and culture maintained at 37°C and 95% air/CO_2_. These microcapsules were similar but not identical to those used in early pilot clinical trials in T1D (14). In fact, here the longer exposure of the microbeads to sodium citrate resulted in better aggregation of the enveloped cells, and eventually facilitated the microcapsules breakage to retrieve the cells.

Microencapsulated cells (CpS-hUCMS) were exposed overnight to Interferon-γ (IFN-γ) (2400U/10^6^ cells) (Sigma-Aldrich) that was removed prior to starting the co-culture incubation system with PBMCs. Cps-hUCMS were tested for viability, after overnight priming with IFN-γ, by ethidium bromide and fluorescein diacetate (Sigma-Aldrich) under fluorescence microscopy, using appropriate filter sets.

### 2.6 Cps-hUCMS viability assay.

The cell viability assay was performed after microencapsulation and after o/n incubation with and without the addition of IFN-γ to verify cells survival within the microcapsules. On this purpose, a mixture was prepared containing Ethidium Bromide 1x (0.2mg/mL) in PBS 1x, to highlight dead cells, and Fluorescein Diacetate in acetone (5mg/mL), to assess the presence of live cells.

### 2.7 Mechanical disruption of the microcapsules and analysis of IDO1 production

Upon o/n culture, after the microencapsulation procedure, two microcapsules aliquots were employed to extract total RNA and proteins. Capsules were resuspended in 4mL of saline and transferred onto a 6-well multiwell plate. Inside the well, the capsules were mechanically broken by a 22G syringe. Thereafter, the solution containing the aggregates was recovered and filtered through a 180-µm mash so that the aggregates (50-150µm in diameter) were eluted within the tube, while the residue was retained by filter. The recovered cell aggregates were washed with saline and subjected to RNA or protein extraction.

### 2.8 Transcriptional expression analysis by quantitative PCR

Total cellular RNA was extracted from the cells (hUCMS or PBMC) using Direct-zol (Zymo Research corp). cDNA was synthesized using the iScript cDNA Synthesis Kit (Bio-Rad Laboratories) and was used as a template for quantitative PCR (qPCR). qPCR primers were designed using sequences from GenBank (http://www.ncbi.nlm.nih.gov/Genbank) (see **supplementary material**). qPCR amplifications were performed using the SsoAdvanced Universal SYBR Green Supermix (Bio-Rad Laboratories) (RRID : SCR_008426) and Agilent AriaMx (RRID : SCR_019469) (Stratagene, La Jolla, CA). PCR products were demonstrated to be a single PCR product, by melting curve and electrophoresis analysis.

### 2.9 Western blotting

Protein samples (40µg) were analyzed on 10% SDS-PAGE and transferred onto nitrocellulose membrane (Bio-Rad Laboratories). The used detection primary antibody was mouse anti‐human IDO1 (1:10000, Millipore). Immunodetection was performed by Clarity Chemiluminescent kit (Bio-Rad Laboratories).

### 2.10 Cytokine assay

Supernatants taken from the co-cultures of two representative healthy subjects while two representative SS patients were selected for simultaneous analysis of 15 cytokines (IL1a, IL1b, IL2, IL4, IL5, IL6, IL10, IL12, IL13, IL15, IL17, IL23, IFNγ, TNFα, TNFβ) by the Multiplex method. For each condition, 50µL of supernatant pre-diluted 1:2 in triplicate was used.

### 2.11 Statistical analysis

Data were graphically represented and analyzed with GraphPad Prism (GraphPad Software) (RRID : SCR_002798), and Mann-Whitney, Wilcoxon. ANOVA tests were applied where necessary. In the evaluation of two single groups the unpaired Student t-test was performed. All statistical tests were two-tailed, and values of p<0.05 were considered statistically significant. qPCR reactions were performed in triplicate and intra-assay variance was considered acceptable when SD was lower than 0.5. The results obtained have been expressed as ‘‘fold changes’’. HPRT1 served for control. All results were expressed as mean ± SEM of at least three independent experiments (*p<0.05). The exploratory study design did not require a power analysis to determine sample or group size.

## 3 Results

### 3.1 Clinical characteristics of the selected SS patient group

The cohort included 9 female SS patients with a median age of 58 (49–68) years and a median disease duration of 2 (1,5-14,5) years. No dropouts or loss of samples and subjects were registered. As expected, xerophtalmia and xerostomia were the most frequent symptoms characterizing the whole cohort, both being reported in 83% of patients. History of parotid swelling was reported in 1 (17%) patient. Inflammatory articular involvement was the most frequent extra-glandular manifestation (33%), followed by vasculitic purpura (17%) and Raynaud phenomenon (17%). As far as serologic features are concerned, anti-Ro antibodies were positive in all patients and half of them were also positive for anti-La antibodies. Two (33%) patients displayed serum positivity for rheumatoid arthritis factor and 1 (17%) had positive cryoglobulins without features suggestive for active cryoglobulinemia. Finally, minor salivary gland biopsy resulted positive (focus score ≥ 1) in all patients. Hemocytometry analysis, performed in conjunction with blood sampling, showed normal white blood cell count in all patients and controls. Basal Lymphocyte Counts

Counts of the immune cell subsets of interest in peripheral blood of SS patients and controls were performed by the time of collection (**Table 1**). Data were consistent with what had been described in the literature and attributable to either recruitment of immune cells into the exocrine glands, or the inflammatory condition typical of the disease (15, 16). In fact, in the selected SS patients, lymphocytes/μl were significantly lower than controls. Moreover, the frequency of Tang, Th17DN, Breg and was lower than in healthy people; the frequency of CD19^+^ B lymphocytes showed no differences.

**Table 1:** Frequency (no. cells/µL) of immune subsets in the peripheral blood of the group of healthy subjects (CTRL) and patients with SS (PZ SS) at the time of collection.

Subpopulations	CTR (6)	PZ SS (9)	P value (significantly different p<0.05)
**Mean lymph/μL**	1833 ± 378	1300 ± 126	0.0083
**Lymphocytes T CD3+**	1391 ± 205	809 ± 104	0.0002
**Lymphocytes Tang**	403 ± 199	156 ± 61	0.0176
**Lymphocytes Th17 DN**	103 ± 16	53 ± 24	0.0056
**Lymphocytes B CD19+**	262 ± 69	177 ± 101	No
**Lymphocytes Breg**	25 ± 6	8 ± 3	0.0011

Values are expressed as mean ± S.D.

### 3.2 Cps-hUCMS analysis, proliferation and eTreg

#### 3.2.1 CpS-hUCMS analysis

**Figure 1:**
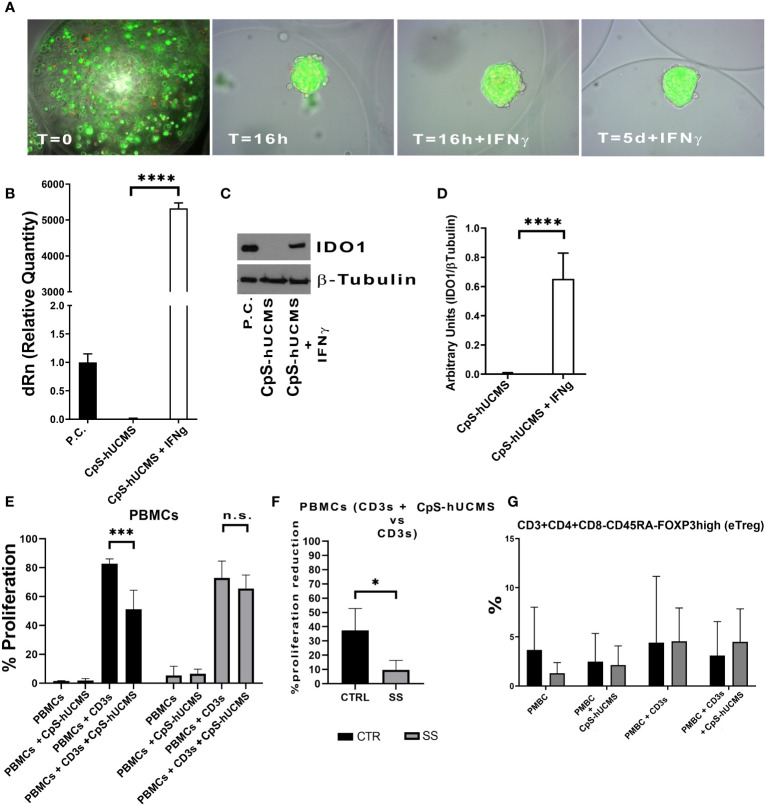
**(A)** Microcapsules containing hUCMS after the procedure and at the indicated times, ethidium bromide/fluorescein assay demonstrates the optimal viability of single cells and aggregates over the experiment duration. **(B)** Expression levels of IDO1 as mRNA and as protein **(D)** in CpS-hUCMS and CpS-hUCMS + IFNγ in comparison to a positive control. **(C)** Representative autoradiographic plate of the expression of IDO1 (45kDA) and tubulin (50kDA) in the indicated samples is shown. **(E)** Proliferation percentage of PBMCs from the group of healthy subjects (CTR) and SS patients (SS) in the various conditions (mean ± S.D., Anova Test, p<0.001, n.s. not significant). **(F)** Percentage reduction in PBMCs proliferation of recruited patients and controls (mean ± S.D., Mann-Whitney T-Test). **(G)** eTreg in control vs SS at the indicated conditions. Biological variability between samples is evident, but it is clear that PBMCs from SSs after 5 days of culture show much lower percentages of eTreg than controls while the presence of Cps-hUCMs increases their percentage.

hUCMS formed cell aggregates upon microencapsulation in AG (9, 10). **Figure 1A** shows microcapsules containing hUCMS, fresh and upon overnight incubation. Within microcapsules, the cells formed compact 3D aggregates measuring 50 to 150 μm in diameter. A few cells failed to aggregate, and remained disperse within the microcapsules. Under fluorescence microscopy, upon staining with ethidium bromide and fluorescein diacetate, both disperse and clustered cells looked very viable. Aggregate formation, induced by the capsule citration procedure (to remove the capsular gel core), combined with optimal cell viability, is essential for both, cells survival in culture for 5 days (**Figure 1A**) and optimal IDO1 production in response to IFNγ. Specifically, we confirmed that IFN-γ concentration of 2400 U/10^6^ cells, previously used in our experiments (9), greatly enhanced production capacity of IDO1 in response to IFNγ concentrations from microencapsulated hUCMS. In fact, it was possible to evidence both, messenger induction and protein production (**Figures 1B, C**). Furthermore, WB consistently showed production of the IDO1 protein after o/n priming with IFNγ in all experiments (**Figure 1D**).

#### 3.2.2 Inhibition of proliferation evaluation

At the end of the five-day incubation period with and without CpS-hUCMS, PBMCs from controls and SS patients were assessed cytofluorimetrically to assess proliferation levels (**Figure 1E**). Within our control samples, we observed an average proliferation increase of 81% ± 11 in PBMCs activated with CD3s as compared to those maintained in simple medium; PBMCs activated and incubated with CpS-hUCMS showed a decrease in proliferation rate to around 51% ± 12. In **Figure 1E**, the proliferative rates of PBMCs in complete medium and with CpS-hUCMS are also compared, with the latter showing no proliferation stimulation in control PBMCs.

PBMCs from SS patients also showed a behavior similar to that of healthy subjects; however, activation with CD3s induced a similar proliferative response (mean value 78 ± 5%) whereas the presence of CpS-hUCMS was associated with a weaker decrease in proliferation (72 ± 2%) (**Figure 1F**). Thus, we observed a greater inhibitory effect in the controls (37.1 ± 16.52%) than in the patient samples. In fact, the percentage of inhibition of PBMCs in SS patients was significantly lower than that of controls (9.79 ± 6.55%). The high reactivity and inflammatory nature of SS PBMCs could explain these readings but, nevertheless, CpS-hUCMS were shown to inhibit their proliferation.

#### 3.2.3 Treg characterization

**Figure 2:**
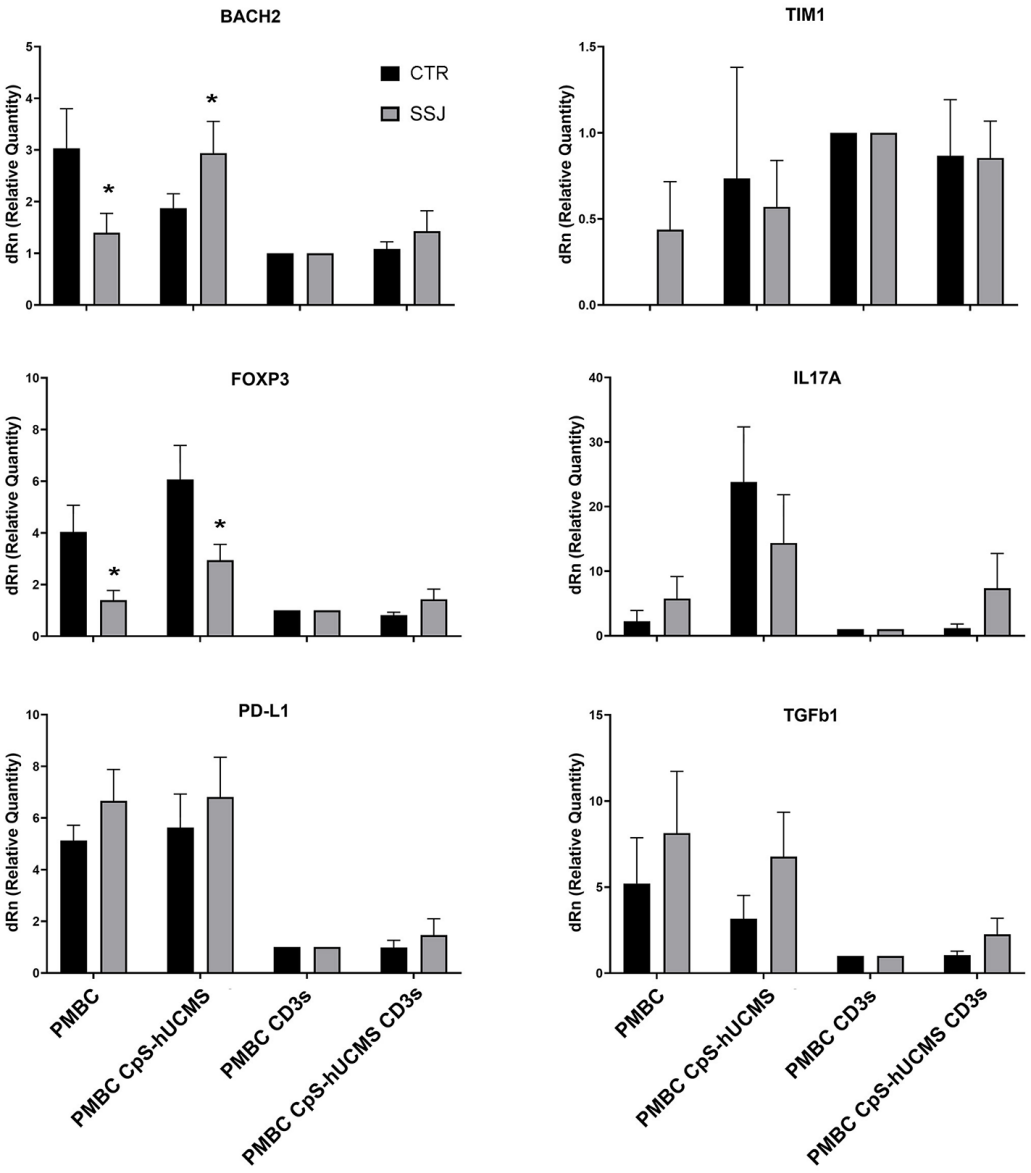
qPCR for indicated messengers expressed byPBMC of control and Sjogren patients after 5 days of culture with or without CpS-hUCMS (pretreated with IFNγ 2400 U/10^6^ cells) and with or without CD3. The expression of each marker was calculated by assigning PBMC+CD3s with the arbitrary value 1. HPRT1 served for control. All results were expressed as mean ± SEM (*P<0.05).

We observed the presence of an increase in Treg by characterizing them as CD3^+^CD4^+^FOXP3^+^ cells and used the marker CD45RA in order to distinguish effector Treg (eTreg CD4^+^CD45RA^-^FOXP3^+^) from naive Treg (nTreg CD4^+^CD45^+^FOXP3^LOW^) and from non-Treg FOXP3^+^ cells. Both nTreg and eTreg were low in SS after 5 days of culture, as compared to controls. However, upon CpS-hUCMS exposure, eTreg in PBMCs from SS equaled controls (**Figure 1G**). Moreover, the FOXP3 messenger in these PBMCs was basally much lower as compared to controls, but its relative expression doubled after co-culture with CpS-hUCMS (**Figure 2**). However, the presence of CpSs did not affect nTreg.

### 3.3 T lymphocytes characterization

#### 3.3.1 Angiogenic T lymphocytes

Angiogenic T lymphocytes (Tang) constitute a recently discovered immune subset identified by the CD3^+^CD31^+^CD184^+^ phenotype (17) and involved in the repair of vascular damage and angiogenesis through release of soluble factors and the mobilization of endothelial progenitor cells. For this reason, characteristics of this T subset and its response to CpS-hUCMS were investigated.

**Figure 3:**
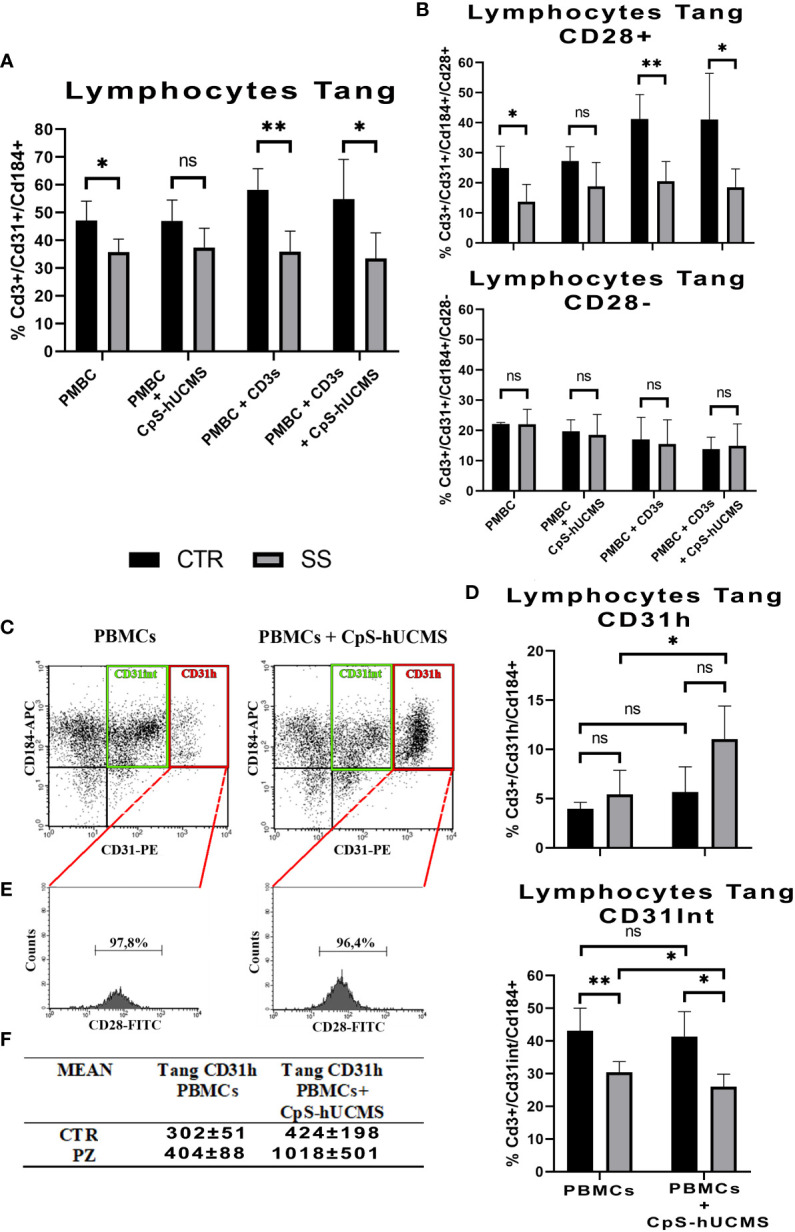
**(A)** Comparison of the percentages of CD3^+^CD31^+^CD184^+^ Tang lymphocytes in the PBMCs between healthy subjects (CTR) and SS patients (SS) under different conditions (mean ± S.D., Mann-Whitney T-Test, * p<0.05, ** p<0.005). No statistically significant differences were found in Tang lymphocyte percentages between conditions within the control and patient groups. **(B)** Percentage of CD28^+^ and CD28^-^ Tang lymphocyte subsets within the total Tang lymphocyte population of healthy subjects and SS patients at the different conditions. (mean ± S.D., Mann-Whitney T-Test, p<0.05*, p<0.005*, n.s. not significant). No statistically significant differences were found in the percentages of CD28^+^ and CD28^-^ Tang lymphocytes between conditions in the control group and in the patient group, with the exception of the comparison between control PBMCs treated with soluble anti-CD3 and PBMCs regarding CD28^+^ (p<0.05). **(C)** Representative cytofluorimetric dot-plots of total Tang lymphocytes belonging to a SS patient after five days of incubation in the absence (left) and presence (right) of CpS-hUCMS. The population in the red rectangle was called CD31^H^ Tang; while that in the green rectangle is the Intermediate Tang. **(D)** Comparison of the percentages of Tang CD31^H^ and Tang CD31^Int^ lymphocytes in the total Tang CD3^+^CD31^+^CD184^+^ of healthy subjects (CTR) and SS patients (SS) in PBMCs and in PBMCs maintained in the presence of CpS-hUCMS. Significant was the increase in CD31^H^ in PBMCs from SS in the presence of CpS-hUCMS. (mean ± S.D., Mann-Whitney T-Test & Wilcoxon T-Test, p<0.05*, p<0.005*, n.s. not significant). **(E)** Representative percentage of CD28 in the CD31^H^ in selected SS patient. Almost all CD31^H^ also expressed CD28. **(F)** Table with averages of CD31^H^ in controls and patients with and without CpS-hUCMS.

Comparison of the two study groups after 5 days of incubation under the various experimental conditions, was associated with a significantly higher percentage of Tang lymphocytes in controls than in patients, under all conditions, except when PBMCs were exposed to CpS-hUCMS. This may suggest that hUCMSC exert the modulating effect equally, in healthy subjects and patients (**Figure 3A**). No significant differences in Tang lymphocyte levels were found between conditions in each group. This finding may suggest an impairment of the vascular repair mechanisms in patients, that is corrected by CpS-hUCMS.

#### 3.3.2 CD28^+^ T angiogenic and CD28^-^ T angiogenic

CD28 represents a key cell surface marker for sorting Tang lymphocytes into two different subsets (18, 19): CD28^+^ tang and CD28^-^tang (**Figure 3B**). The former hallmarks younger cells that are actively involved in the process of angiogenesis through the release of cytokines such as IL8, IL17 and VEGF; the latter identifies a senescent phenotype. In the PBMCs of CTRs, there is an approximately equal percentage of CD28^+^ and CD28^-^ Tang, and the presence of CD3s or CD3s with CpS-hUCMS results in a shift towards the CD28^+^ subset (PBMCs+CD3s: 41,3 ± 8,1%; PBMCs+CD3s+CpS: 41,1 ± 15,4%). In SS samples, the Tang after 5 days of culture are mostly CD28; however, the presence of CpS-hUCMS, CD3 with and without CPS-hUCMS, results in an increase of CD28^+^ Tang cells such that the percentages observed in the control samples are restored.

#### 3.3.3 Angiogenic T subset: Tang CD31^H^

Cytofluorimetric dot-plots of the Tang CD3^+^CD31^+^CD184^+^ T relative to the PBMCs of the controls and especially of the SS in the presence of the CpS-hUCMS (**Figure 3C** indicated the emergence of a particular angiogenic lymphocyte subset, to our knowledge never previously described in the literature, characterized by a high expression of CD31 and definable as Tang CD31^H^, and an angiogenic T subset encompassing Tang Intermediate (Tang CD31^Int^) cells, with intermediate expression levels of CD31). Under activation conditions (presence of CD3s), the Tang CD31^H^ population is not detectable within the PBMCs in either group, as confirmed by the published literature (20). **Figure 3D** shows the comparison between Tang CD31^H^ and Tang CD31^Int^ of controls vs. SS, with and without CPS-hUCMS. PBMCs from SSs showed a significant duplication of % Tang CD31^H^ within PBMCs co-cultured with CpS-hUCMS, as compared to cells incubated in medium only. The Tang CD31^Int^ of controls were higher in percentage than those of SS, and were not adversely affected by the presence of CpS-hUCMs, whereas, with respect to SS, percentage reduction of Tang CD31^Int^ in the presence of hUCMSC was statistically significant. Hence in SS, CpS-hUCMS resulted in significant increase in Tang CD31^H^ (**Figure 3F**) and this was due to the increase of CD31 expression by Tang CD31^Int^. Furthermore (**Figure 3E**) over 95% of the Tang CD31^H^ T were CD28^+^ both, in the presence and absence of the CpS-hUCMS in both groups. Collectively, these observations allowed us to define a new, previously undescribed T cells population, characterized by the high expression of CD31 and positive for CD28, which warrants further study.

### 3.4 B lymphocytes and B regulatory lymphocytes

#### 3.4.1 CD19^+^ B lymphocytes

**Figure 4:**
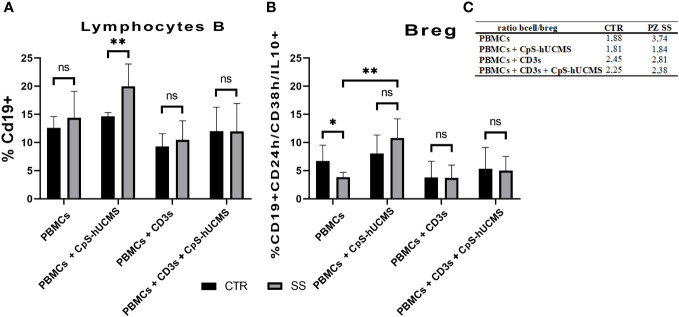
**(A)** Proportion of CD19+ B lymphocytes in PBMCs of healthy subjects (CTRL) and SS patients (SS) in the various conditions (mean ± S.D.; Mann-Whitney T-Test; p<0.005**; ANOVA test in SS; p<0.005** between CpS-hUCMS vs Activated and CpS-hUCMS vs Act. + CpS-hUCMS). **(B)** Percentage of Breg lymphocytes in the PBMCs of healthy subjects (CTR) and SS patients (SS) in the various conditions (mean ± S.D.; Mann-Whitney T-Test; p<0.5*; ANOVA test in SS; p<0.005** between Medium vs CpS-hUCMS, CpS-hUCMS vs Activate and CpS-hUCMS vs Att. + CpS-hUCMS). **(C)** B/B-reg lymphocytes ratio in the two groups at various conditions.

B lymphocytes are crucial for the progression of SS: they intervene during the most advanced stages of the disease and directly or indirectly promote inflammation through the production of autoantibodies. Given their relevance in the disease and the lack of data on the effects of CpS-hUCMS on this cell population, we first examined CD19^+^ B lymphocytes. Obtained data (**Figure 4A**) showed that in SS PBMCs co-cultured with CpS-hUCMS, there was a significant higher increase in the percentage of CD19^+^ B lymphocytes than in controls (CTR: 14.67% ± 0.33; SS: 19.97 ± 1.6; p=0.0095**). In contrast, no clear variations between healthy versus unhealthy were found within the other conditions.

In the control group, there were no differences in the percentage of CD19^+^ B-cells between conditions, whereas in the SS group, a significant difference was found between the CD19^+^ number in CpS-hUCMS co-cultures and that of activated PBMCs both in the presence/absence of encapsulated mesenchymal cells. This finding is partly explained by the decrease of B-cells when an activating stimulus such as CD3s is introduced into the system. The qPCR (**Figure 2**) for transforming growth factor-beta 1 (TGFβ1) messenger confirmed the trend described for CD19^+^ B in the two groups. In fact, it is on average more highly expressed in the PBMCs of SS patients than in controls, both grown in basal medium and in the presence of CpS-hUCMS. The presence of CD3s downregulates its expression in both groups. PBMCs from SS grown in medium for 5 days showed lower levels of messenger for BACH2 (**Figure 2**) as compared to control PBMCs, whereas in the presence of CpS-hUCMS PBMCs from SS, showed higher levels for this messenger than control PBMCs. This messenger was also inhibited by the presence of CD3s, and CpS-hUCMS was irrelevant.

Furthermore, the expression of CD20^+^, a marker of B lymphocyte maturation progression, indicated that there were no significant differences between either study groups or conditions (data not shown). This finding seems to indicate that although CpS-hUCMS stimulated CD19^+^ B lymphocyte proliferation, it did not allow them to reach full maturity.

#### 3.4.2 B10 regulatory lymphocytes

We aimed to evaluate the effects of microencapsulated hUCMS on regulatory B10 lymphocytes, i.e., those expressing the anti-inflammatory cytokine IL10. In particular, we studied the ‘transient’ Breg subset, characterized by CD19^+^ cells predominantly positive for IL10 and expressing high levels of the surface markers CD24 and CD38. The co-culture experiments showed that there was a significant difference between the percentage of Breg B10 (**Figure 4B**) in healthy subjects and SS patients cultured in medium alone. In the SS group, the presence of the CPS-hUCMS led to significant increase in this subset as compared to all other conditions, with a restoration of the Breg cell subset to the levels found in healthy individuals. Activation with CD3s depressed this population and the concomitant presence of CPS-hUCMS increased their percentages in both controls and SS to pre-activation levels.

### 3.5 Cytochines’ evaluation

**Figure 5:**
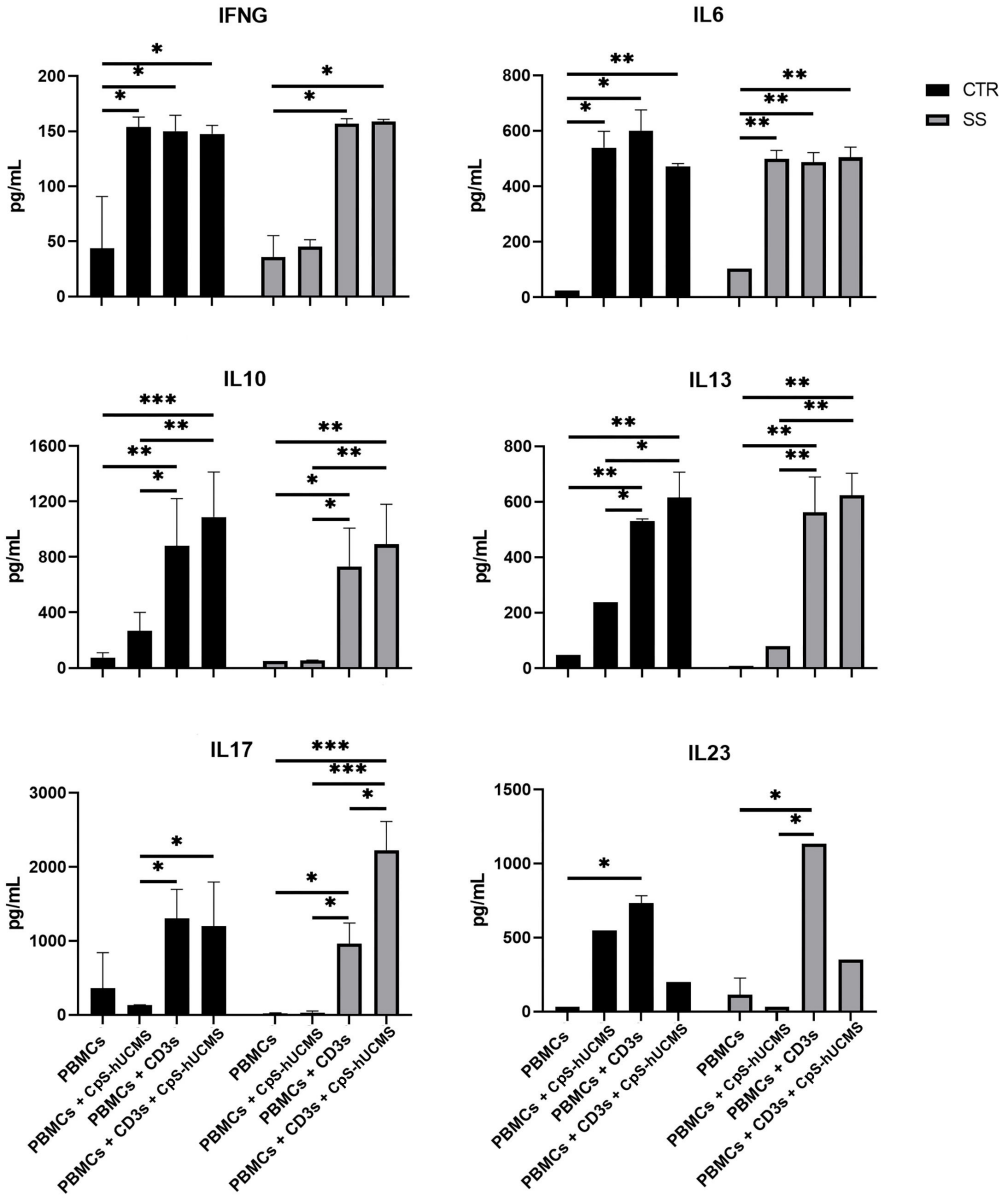
Amount of indicated cytokines (pg/mL) released in the supernatant from PBMC of control and Sjogren patients after 5 days of culture with or without CpS-hUCMS (pretreated with IFNγ 2400Y/10^6^ cells) and with or without CD3s. All results were expressed as mean ± S.D. (*P<0.05, **P<0.005, ***P<0.0005).

The data obtained by measuring the concentration of certain cytokines and IFNγ (**Figure 5**) in the supernatant of the co-cultures and the expression of specific messengers (**Figure 2**) in the PBMCs helped us to better understand the action of stem cells on Tang. In particular, CPS-hUCMS in PBMCs from SS led to a lower concentration of IL17 and IFNγ in the culture medium as compared to controls. Activation with CD3s increased concentration of all cytokines leading, in controls, to maximum concentration which was negatively modulated by the presence of CpS-hUCMS, as far as IL6 and IL23 but not IL17 and IFNγ were concerned. In SS, CD3s increased their concentrations; for IL6 and IFNγ the simultaneous presence of CpSs was irrelevant; for IL23 the presence of CpSs and CD3 induced a marked decrease comparable to control, whereas IL17 doubled its concentration as compared to simple activation with CD3 and compared to the respective control points. qPCRs for the IL17A messenger (**Figure 2**) showed an increase in its relative amount in PBMCs from SS in the presence of CpS-hUCMS, which for control PBMCs was more evident but, as described above, it was not accompanied by a similar increase in the concentration of this cytokine in the culture medium.

Further evidence confirming the modulatory activity of CpS-hUCMSs on Breg lymphocytes came from the amount of IL10 detected in the supernatant under various conditions (**Figure 5**). In SS patients, unlike healthy subjects, the presence of CpS-hUCMSs alone did not lead to an increase in the production of this cytokine, which instead reached high levels once upon CD3s introduction. This finding suggests that CpS-hUCMS stimulate the regulatory component of B lymphocytes, to which we attribute the increased expression of this cytokine, not only in terms of proliferation but also of production of IL10, which would be stored within the cytoplasm. However, whereas in healthy individuals there is a discrete increase of IL10 in the supernatant of the co-cultures, in SS patients the presence of an additional stimulus (in our case CD3s) is required for the complete release of IL10. The increase in Breg CD19^+^CD24^+^CD38^+^IL10^+^ induced by CpS-hUCMSs occured at expense of reduction of B CD19^+^, which brought the Bcell/Breg ratio up to that of controls (**Figure 4C**).

### 3.6 Messangers’ evaluation

PD-L1 a receptor expressed on T and B cells, upon activation, is able to inhibit proliferation or activate the process of cell death of lymphocytes: in fact, in our samples after activation with CD3s the expression of its messenger declined in both controls and patients with SS (**Figure 2**). Its trend is similar to that of TGFβ1. The messenger for TIM1 on control PBMCs was never expressed; in some subjects it appeared after co-culture with CpS-hUCMS, resulting in wide variability in these samples; it is then well expressed after stimulation with CD3s with and without CpS-hUCMS. In contrast, in patients with SS it was present on PBMCs cultured in simple medium and in the presence of CpS-hUCMS and up-regulated after stimulation. TIM1 encodes for a glycoprotein expressed on the surface of Th2 lymphocytes that is involved in T-cell activation, being also present on Breg, induced by mesenchymal stem cells.

## 4 Discussion

The most important finding of this work can be summarized as follows: enhancement of Tang CD28^+^ lymphocyte subset, definition of new CD31^H^ angiogenic T lymphocyte subset, and increase of B10 regulatory subset (Breg CD19^+^CD24^H^CD38^H^IL10^+^), all the above described effects are attributable to the action of CpS-hUCMS.

We previously (9) demonstrated that CpS-hUCMS represents a functional biohybrid artificial system where cellular/molecular products induce powerful immunomodulatory *in vitro* effects on Treg cells of patients with SS. Instead now the aim of the present work was to investigate the effects on other Tc populations and also on B cell subsets with a particular focus on regulatory B cells. hUCMS are associated with important immunomodulatory effects through both, cell-to-cell contact and release of soluble factors (TGFβ1, IDO, HLAG5 IL6, PGE2, VEGF). Many studies in the literature confirm the beneficial effects of their application to various diseases, however some of them (3, 21–23) conducted *in vitro*, have also shown how autoimmune PBMCs can reduce the functionality of mesenchymal stem cells upon direct contact. As we have already pointed out (11), very useful is the use of a polymeric artificial matrix, e.g. endotoxin-free alginate, to prevent direct contact of hUCMS with the host’s immune system, although with no interference with secretion of immunomodulatory molecules. Most importantly, sodium alginate microcapsules, when properly formulated, allow hUCMS to acquire a three-dimensional structure (9) that allows them to survive longer in comparison with not aggregated cells, and enhances secretion of various immunomodulatory factors (24) after treatment with IFNγ. Data on viability, IDO1 production and ability to inhibit proliferation of stimulated PBMCs confirm the effects of CpS-hUCMS on patient PBMCs. In addition, hUCMS have been shown to affect preferentially eTreg, by increasing their rates, which enabled us to complete our previous observations (9).

### 4.1 Enhancement of Tang CD28^+^ lymphocyte subset

The effects of Cps-hUCMS on Tang is remarkable (17). This is a lymphocyte subset that reacts to vascular wall injuries and induces, in cooperation with endothelial cells (EPCs) and other immune populations (Treg), tissue repair, with the involvement of cytokines and pro-angiogenic factors (IL8, IL17, VEGF) (18). They are also endowed with a high capacity of adhesion to ECs and trans-endothelial migration. Altogether, these properties give Tangs the ability to promote formation of new vessels *in vitro* and *in vivo* (17). Recent literature shows that the percentage of Tang lymphocytes in the peripheral circulation is markedly reduced in subjects with SS, leading to defective vascular layer restoration and endothelial cells dysfunction. This data supports previous reports (17, 25–27). Tang can be classified according to the presence/absence of CD28 (18), one of the two known ligands for CD80/86, a cell surface molecule involved in T-cell activation, in the induction of cell proliferation, cytokine production and of T-cell survival promotion. It should be noted that CpS-hUCMS did not affect Tang cell proliferation but changed the ratio within the same population, thereby favoring the immature and more active cell phenotypes (Tang CD28^+^) (28) and restoring a situation similar to controls. Decrease in Tangs CD28^-^ in our patients is desirable because this population correlates directly with serum levels of cytokines and autoantibodies associated with endothelial damage and poor SS prognosis (28).

### 4.2 New CD31^H^ angiogenic T lymphocyte subset

In addition, analysis of Tang lymphocytes, (CD3^+^CD31^+^CD184^+^), showed the presence in the co-cultures with PBMCs of both, controls, and SSs of a defined CD31^H^ angiogenic T lymphocyte subset that was never previously described in the literature. The presence of CpS-hUCMS (probably through production of VEGF and IL6, two potent activators of angiogenesis) results in a significant increase in the percentage of CD31^H^ Tang in SS, as does the reduction that occurs within Tang CD31^Int^. The fact that almost all CD31^H^ Tang cells express CD28 makes this subset numerically not negligible. The enhanced action of Tang CD28^+^ expressing large amounts of CD31 by hUCMS could have very positive impact on patients. CD31 (29) is an efficient signaling molecule that plays several roles in vascular biology including angiogenesis, platelet function, and thrombosis, mechano-sensing of endothelial cells in response to fluid shear stress, and regulation of multiple stages of leukocyte migration through venular walls. Chronic autoimmune diseases are associated with increased risk of cardiovascular death, and endothelial dysfunction represents the first stage of subclinical atherosclerosis (25). In SS, an increment of new blood vessel formation associated with an increased number of macrophages and histiocytes infiltrating in the stroma of the inflammatory lesions may occur (25) but it is a defective venous system that can be healed by young Tang with the higher expression of CD31 induced by CpS-hUCMS (30).

**Figure 6:**
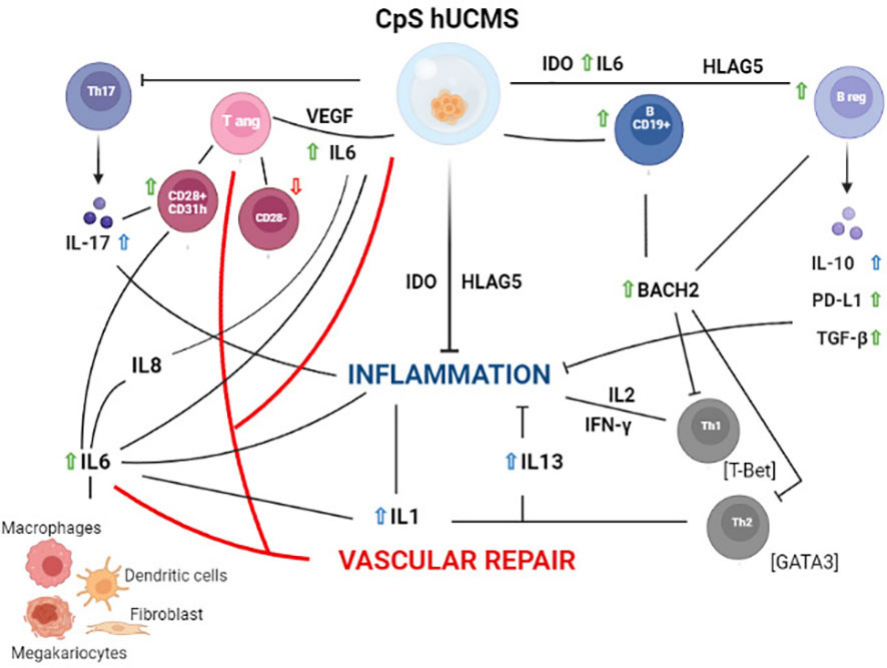
Schematic representation of the hypothetical mechanisms of action of PBMC from Sjogren patients after 5 days of culture with CpS-hUCMS. Green arrows indicate an increase in the presence of CpS-hUCMS; red arrows indicate a decrease in the presence of CpS-hUCMS; whereas arrows in blue indicates an increase in the presence of both CpS-hUCMS and CD3s.

Based on our experimental evidence, we can hypothesize (**Figure 6**) that our findings occur as a consequence of certain soluble factors such as IL6, IL17 and IFNγ, which are present in high amounts in the supernatant of co-cultures. IL6, in addition to being produced by different lymphocyte types, is included in the secretome of hUCMS (31–34) and is responsible, together with VEGF, for maintaining the inflammatory and pro-angiogenic microenvironment. Once released, IL6 would act in our system on CD28^+^ Tang forcing them to produce IL8, IL17 and VEGF (35) hence creating a pro-inflammatory and angiogenic circuit in the vascular microenvironment aimed at restoring its functional integrity.

### 4.3 B10 regulatory subset (Breg CD19^+^CD24^H^CD38^H^IL10^+^)

The results obtained from the analysis of the B lymphocyte population subset after five-day incubation with CpS-hUCMS showed how the latter are able to condition this cell type in terms of both, proliferation, and function. CD19^+^ represents a characteristic maturation marker of the B lymphocyte lifespan: when these cells take over the plasma cell phase, they tend to lose it, as we saw after the addition of CD3s to the system. The various soluble stem cell factors (VEGF, IL6, HLAG5, IDO1) seem to prevent this process, by keeping the B cells in a state of functional immaturity and stimulating the Breg lymphocytes to produce IL10, PDL1, and TGFβ1 before spilling them into the surrounding environment. This event occurred with greater effects in SS samples than in CTRs.

Another evidence in support to the effects of CpS-hUCMS on B lymphocytes comes from the mRNA expression of BACH2, a transcription factor that, through Treg differentiation and maintenance, promotes B cell proliferation and differentiation into memory cells. This protein acts on two stages of B lymphocyte maturation: within the thymus, high expression of BACH2 guides the cells to various maturation stages, by promoting the expression of specific surface markers; in the periphery, however, BACH2 is gradually down-expressed until, having reached the plasma cell stage, it is no longer produced. If we consider the high levels observed in SS and CTRLs when PBMCs were co-cultured with CpS-hUCMSs and compared them with those when CD3s were added, it can be assumed that stem cells prevent B lymphocytes from continuing on their pathway to the plasma cell stage to keep them in an immature, quiescent state.

The action of CpS-hUCMS also seems to extend to the B10 regulatory subset (Breg CD19^+^CD24^H^CD38^H^IL10^+^), leading especially in SS to an important percentage increase of this population, as compared to that recorded for PBMCs alone. Molecular analysis showed reduced expression of the messenger for BACH2 in PBMCs, and increased expression of PD-L1 and TGFβ1, which are crucial for the Breg regulatory activity (36, 37). Since, unlike what was observed in controls, the Breg of SS samples seem unable to release IL10 in the presence of CpS-hUCMS we might assume that the Breg of SS patients are characterized by functional defects (38), and that they need the joint stimuli of CpS-hUCMS, CD3s and the Tc in order for them to release the IL10 stored in the cytoplasm. This regulatory system that would be activated by CpS-hUCMS could contribute to modulate the pro-angiogenic circuitry thus preventing the inflammation to become uncontrolled and result in further damage.

We may then hypothesize that CpS-hUCMS result in a blockade of the maturation of B lymphocytes into plasma cells and their redirection toward the regulatory phenotype (39). In particular, other soluble mediators abundantly produced by mesenchymal stem cells could be responsible for these effects: IDO1 and HLAG5 are important in inhibiting immune activation and promoting the differentiation of regulatory subsets of T and B lymphocytes (40); IL6, possibly associated with IL1β, for induction of Breg lymphocytes (41). Finally, VEGF, which not only stimulates vascular repair but also promotes the survival and proliferation of peripheral CD19^+^ B lymphocytes through blocking caspase 3-mediated apoptosis (42).

## 5 Conclusion

In this work we provided preliminary evidence that CpS-hUCMS represent a functional biohybrid artificial system where cellular molecular products are able to exert powerful immunomodulatory effects *in vitro* on T cells and B cells in pSS. In particular, we described the strong induction of Breg lymphocytes and the emergence of a Tang phenotype greatly expressing CD31. Both will deserve to be studied in depth, by subtyping and function assays, in order to confirm the postulated beneficial action on patients.

## Data availability statement

The original contributions presented in the study are included in the article/**Supplementary Materials**, further inquiries can be directed to the corresponding author/s.

## Ethics statement

The studies involving human participants were reviewed and approved by local ethical committee for clinical studies (CEAS). The patients/participants provided their written informed consent to participate in this study.

## Author contributions

PM designed the research, performed experiments, analyzed data, write the article, arranged figures, OB designed the research, performed experiments, write the article, MA performed experiments, analyzed data, write the article, TP cultured hUCMS, performed experiments, AG performed experiments, GB performed microencapsulation, EB patients’ enrollment, RG edit the article, RC edit the article and supervised experiments. All authors contributed to the article and approved the submitted version.
