# Supplementary material for: Emerging of a new CD3+CD31HCD184+ tang cell phenothype in Sjögren’s syndrome induced by microencapsulated human umbilical cord matrix-derived multipotent stromal cells
Source: Front Immunol. 2023 Mar 14;14:1095768. doi: 10.3389/fimmu.2023.1095768 (PMC10043489; doi:10.3389/fimmu.2023.1095768)
Supplement: Supplementary file 1 [file Table_1.docx]

Supplementary Material

EMERGING OF A NEW CD3^+^CD31^H^CD184^+^ Tang CELL PHENOTHYPE IN SJÖGREN’S SYNDROME INDUCED BY WITH MICROENCAPSULATED HUMAN UMBILICAL CORD MATRIX-DERIVED MULTIPOTENT STROMAL CELLS.

Pia Montanucci^1†^, Onelia Bistoni^2†^, Matteo Antonucci^3†^, Teresa Pescara^1^, Alessia Greco^1^, Giuseppe Basta^1^, Elena Bartoloni^2,3^, Roberto Gerli^2,3^, Riccardo Calafiore^1*^

Table S1: primers used in qPCR. Primers were designed using the sequences from GenBank (htpp://www.ncbi.nlm.nih.gov/Genbank).

| Gene | Primer Forward | Primer Reverse | bp | Tm (°C) |
| --- | --- | --- | --- | --- |
|  |  |  |  |  |
| HPRT1 | 5’-ggtcaggcagtataatccaaag-3’ | 5’-ggactccagatgtttccaaac-3’ | 250 | 60 |
| PD-L1 | 5’-tgcagggcattccagaaaga-3’ | 5’-accgtgacagtaaatgcgttc-3’ | 87 | 60 |
| TIM-1 | 5’-tctcatttccagcagagcagg-3’ | 5’-aaccagctacagaatctgcca-3’ | 209 | 60 |
| BACH2 | 5’-gaccaagctgatgagccctt-3’ | 5’-gaagcagacagctgcaaatga-3’ | 126 | 60 |
| IL17A | 5’-ccataaccggaataccaatac-3’ | 5’-ccagatcacagagggatatct-3’ | 118 | 60 |
| FOXP3 | 5’-ggacaggccacatttcatg-3’ | 5’-gcactggatttgggaag-3’ | 237 | 60 |
| IDO1 | F-5’gccttgcacgtctagttct | R-5’tcttggagagttggcagtaag, | 120 | 60 |
| IL6 | F-5’acagccactcacctcttc | R-5’cctcaaactccaaaagaccag | 239 | 60 |
| IL10 | F-5’gcctaacatgcttcgagatc | R-5’ggtcttcaggttctcccc | 244 | 60 |
| TGFβ1 | F-5’ggacaccaactattgcttcag | R-5’cgggttatgctggttgtac | 205 | 60 |
|  |  |  |  |  |

**
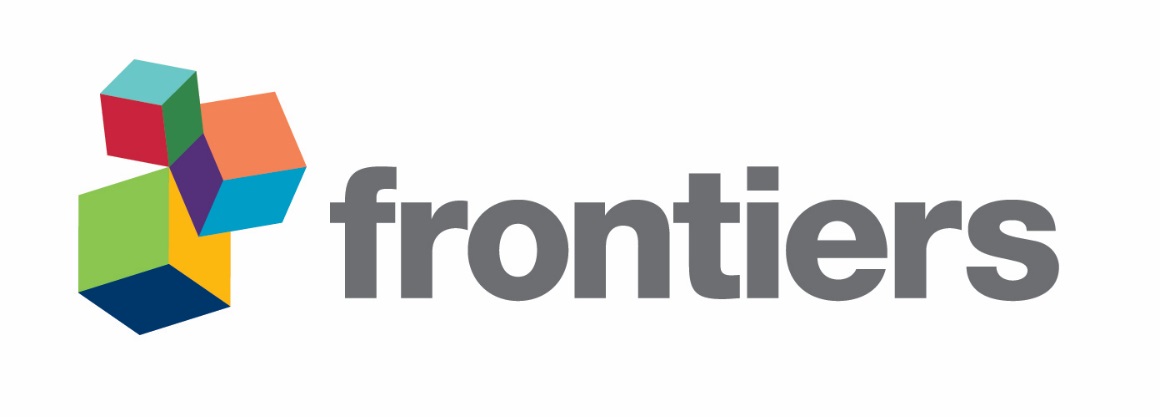
**
